# The alterations of cytokeratin and vimentin protein expressions in primary esophageal spindle cell carcinoma

**DOI:** 10.1186/s12885-018-4301-1

**Published:** 2018-04-02

**Authors:** Xin Min Li, Xin Song, Xue Ke Zhao, Shou Jia Hu, Rang Cheng, Shuang Lv, Dan Feng Du, Xiang Yang Zhang, Jian Liang Lu, Jian Wei Ku, Dong Yun Zhang, Yao Zhang, Zong Min Fan, Li Dong Wang

**Affiliations:** 1 Henan Key Laboratory for Esophageal Cancer Research of the First Affiliated Hospital, Zhengzhou University, 40 Daxue Road, Zhengzhou, Henan 450052 People’s Republic of China; 2 Department of Pathology, Xinxiang Medical University, Xinxiang, 453003 Henan China; 3 Department of Pathology of Basic Medical College, Zhengzhou University, Zhengzhou, 450001 Henan China; 4 Department of Gastroenterology, the Second Affiliated Hospital of Nanyang Medical College, Nanyang, 473061 Henan China; 5 Department of Pathology of Nanyang Medical College, Nanyang, 473061 Henan China; 6 Department of Pathology, Women & Infants Hospital of Zhengzhou, Zhengzhou, 450012 Henan China

**Keywords:** Esophagus, Spindle cell carcinoma, Immunohistochemical staining, Cytokeratin, Vimentin

## Abstract

**Background:**

The accumulated evidence has indicated the diagnostic role of cytokeratin (CK) and vimentin protein immunoassay in primary esophageal spindle cell carcinoma (PESC), which is a rare malignant tumor with epithelial and spindle components. However, it is largely unknown for the expression of CK and vimentin in pathological changes and prognosis of PESC.

**Methods:**

Eighty-two PESC patients were identified from the esophageal and gastric cardia cancer database established by Henan Key Laboratory for Esophageal Cancer Research of Zhengzhou University. We retrospectively evaluated CK and vimentin protein expressions in PESC. Clinicopathological features were examined by means of univariate and multivariate survival analyses. Furthermore, the co-expression value of cytokeratin and vimentin was analyzed by receiver operating characteristic (ROC) curve.

**Results:**

The positive pan-cytokeratins AE1/AE3 (AE1/AE3 for short) staining was chiefly observed in cytoplasm of epithelial component tumor cells, with a positive detection rate of 85.4% (70/82). Interestingly, 19 cases showed AE1/AE3 positive staining both in epithelial and spindle components (23.2%). However, AE1/AE3 expression was not observed with any significant association with age, gender, tumor location, gross appearance, lymph node metastasis and TNM stage. Furthermore, AE1/AE3 protein expression does not show any effect on survival. Similar results were observed for vimentin immunoassay. However, in comparison with a single protein, the predictive power of AE1/AE3 and vimentin proteins signature was increased apparently than with single signature [0.75 (95% CI = 0.68–0.82) with single protein v.s. 0.89 (95% CI = 0.85–0.94) with AE1/AE3 and vimentin proteins]. The 1-, 3-, 5- and 7-year survival rates for PESC patients in this study were 79.3%, 46.3%, 28.0% and 15.9%, respectively. Multivariate analysis demonstrated age and TNM stage were independent prognostic factors for overall survival (*P* = 0.036 and 0.003, respectively). It is noteworthy that only 17.1% patients had a PESC accurate diagnosis by biopsy pathology before surgery (14/82). 72.4% PESC patients with biopsy pathology before surgery had been diagnosed as squamous cell carcinoma.

**Conclusion:**

The present study demonstrates that cytokeratin and vimentin protein immunoassay is a useful biomarker for PESC accurate diagnosis, but not prognosis. The co-expression of cytokeratin and vimentin in both epithelial and spindle components suggest the possibility of single clone origination for PESC.

## Background

The primary esophageal spindle cell carcinoma (PESC), which has also been referred to as carcinosarcoma, sarcomatoid carcinoma, pseudosarcoma, pseudosarcomatous carcinoma, or polypoid carcinoma in literature, has been classified as a subtype of esophageal squamous cell carcinoma by WHO in 2010 [1]. Histopathologically, PESC is characterized by mixed two components, i.e., epithelial and spindle. Although the histogenesis of these two different components remains largely unknown, the accumulated evidence from many case reports has indicated the differential role of cytokeratin (CK) and vimentin protein immunoassay in PESC diagnosis [2–5]. However, it has not been well characterized in terms of immunohistochemical features for CK and vimentin in PESC accurate diagnosis and prognosis prediction.

In the present study, we retrospectively analyzed the CK and vimentin immunoreactivity and their possible roles in accurate diagnosis and prognosis on 82 cases with PSEC, which were retrieved from our esophageal cancer database from 1973 to 2015.

## Methods

### Patients

The two hundreds and eighty-six PESC patients were identified from the esophageal and gastric cardia cancer database (with a total of 500,000 patients) established by Henan Key Laboratory for Esophageal Cancer Research, the First Affiliated Hospital of Zhengzhou University from 1973 to 2015 [6]. Based on the criteria of more than 5 year follow-up after surgical treatment and detailed clinicohistopathological findings, eighty-two PSEC patients were finally enrolled in this study, including 57 males with a mean age of 61.8 ± 8.8 years and 25 females with a mean age of 64.1 ± 6.6 years. All the PESC patients were performed surgical treatment and did not receive any radio- or chemo-therapy before surgery. The clinicopathological characteristics of the 82 patients were summarized in Table 1. The gross appearance of PESC was classified into three types, i.e., polypoid, ulcerative and infiltrating [7]. Overall survival (OS) time was calculated from the day of esophagectomy to death or to the last follow-up. The median follow-up of the entire cohort was 50.1 months (range, 4.1–123.5 months). The success follow-up rate was 94.2%. Informed consent was obtained from all these patients before taking part in our study. This study was approved by the Ethical Committee of Zhengzhou University (No.16047).

**Table 1:** Clinical characteristics of 82 PESC patients n (%)

	No. of case of examination (%)
Age	
< 60 years	26 (31.7)
≥ 60 years	56 (68.3)
Gender	
Male	57 (69.5)
Female	25 (30.5)
Family history	
Positive	18 (22.0)
Negative	64 (78.0)
Smoking	
Yes	41 (50.0)
No	41 (50.0)
Alcohol	
Yes	36 (43.9)
No	46 (56.1)
Tumor location	
Upper^a^	3 (3.7)
Middle	54 (65.9)
Lower	25 (30.4)
Gross appearance	
Polypoid	69 (84.1)
Ulcerative	8 (9.8)
Infiltrating	5 (6.1)
Lymph node metastasis	
Yes	23 (28.0)
No	59 (72.0)
TNM stage	
I	29 (35.4)
II	37 (45.1)
III	16 (19.5)
IV	0 (0.0)

^a^There was one patient whose tumor location was in cervical segment

### Surgical specimen preparation and immunohistochemistry

The entire surgical specimen was routinely formalin-fixed, paraffin-embedded and H&E stained for histopathological diagnosis and immunohistochenistry assay. The immunoreactivty for pan-cytokeratins AE1/AE3 (AE1/AE3 for short) and CK5/6, chiefly in epithelial component and vimentin, chiefly in spindle component, was determined in this study. The AE3 monoclonal antibody recognizes the 65 to 67 triplet, 64, 59, 58, 56, 54 and 52kD proteins also known as cytokeratin 1, 2, 3, 4, 5, 6, 7, and 8 while the AE1 antibody recognizes 56.5, 54, 50, 50, 48, and 40 kDa proteins (also known as CK10, 14, 15, 16 and 19). The AE1/AE3, CK5/6 and vimentin antibodies were all monoclonal mouse antibodies (1:100 dilutions, Gene Tech, USA). Immunohistochemistry was carried out by a two-step protocol (Benchmark XT, Roche). In brief, the 5 μm paraffin-embedded tissue sections were deparaffinized, rehydrated and immersed in 3% hydrogen peroxide solution for 10 min, heated in citrate buffer (pH 6.0) for 25 min at 95 °C, and cooled for 60 min at room temperature. Between each incubation step, the slides were washed with phosphate buffered saline (PBS, pH 7.4). Immunostaining was performed using Roche Benchmark XT with diaminobenzidine (DAB) according to manufacturer recommendations (Gene Tech) and subsequently counterstained with hematoxylin. Slides without the addition of primary antibody served as negative control.

### Assessment of immunohistochemical results

Tissue sections were independently and blindly assessed by three independent histopathologists (XM Li, DY Zhang, and Y Zhang). Discrepancies were resolved by consensus. Positive reactions were defined as those showing brown signals in the cell cytoplasm.

### Statistical analysis

Statistical analysis was performed using SPSS 16.0 software (SPSS Corp, Chicago, IL, USA). Data was represented as the mean ± standard deviation for continuous variables or number (%) for categorical data. Spearman’s two-sided rank correlation and Fisher’s exact test were used to explore the correlation levels between protein expression and clinical characteristics. To estimate the association between eligible variables and survival time, Kaplan-Meier analysis and log-rank tests were used. Univariate and multivariate analyses were based on the cox proportional hazards regression model. Receiver operating characteristic (ROC) curve analysis was used to determine the predictive value of AE1/AE3 and vimentin proteins expression, and the differences in the area under the curve (AUC) were detected by SPSS 16.0. *P* value less than 0.05 was considered statistically significant.

## Results

### AE1/AE3 expression and clinicopathological features

**Fig. 1:**
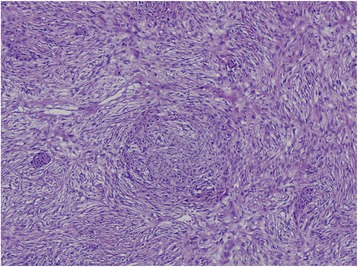
Histological examination (100×) revealed two tumor components

**Fig. 2:**
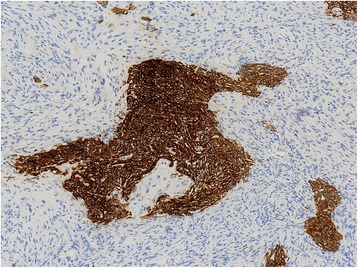
The protein expressions of AE1/AE3 in PESC tissues by immunohistochemistry (100×). The positive immunostaining of AE1/AE3 was localized in cell cytoplasm of epithelial component

Histologically, PESC involve both epithelial and spindle components (Fig. 1). The positive AE1/AE3 staining was chiefly observed in cytoplasm of epithelial tumor cells (Fig. 2), with a positive detection rate of 85.4% (70/82). Interestingly, in 19 cases, AE1/AE3 positive staining was also observed in spindle tumor cells (19/82, 23.2%). However, AE1/AE3 expression was not observed with any significant association with age, gender, family history, smoking, alcohol, tumor location, gross appearance, lymph node metastasis and TNM stage (*P* = 0.070, 0.914, 0.919, 0.755, 0.654, 0.580, 0.660, 0.547, and 0.121, respectively, Table 2). Similarly, considering the AE1/AE3 expression in different components of PESC separately, AE1/AE3 expression showed no correlation with age, gender, family history, smoking, alcohol, tumor location, gross appearance, lymph node metastasis, and TNM stage (*P* = 0.888, 1.000, 0.118, 0.978, 0.764, 0.126, 0.794, 0.449, and 0.146, respectively, Table 3).

**Table 2:** The expression rate of AE1/AE3 protein in PESC

	Number	Expression rate of AE1/AE3 protein (%)		*χ* ^*2*^	*P*
		Positive(*n* = 70)	Negative(*n* = 12)		
Age					
< 60 years	26	19 (73.1)	7 (26.9)	3.28	0.070
≥ 60 years	56	51 (91.1)	5 (8.9)		
Gender					
Male	57	48 (84.2)	9 (15.8)	0.01	0.914
Female	25	22 (88.0)	3 (12.0)		
Family history					
Positive	18	16 (88.9)	2 (11.1)	0.01	0.919
Negative	64	54 (84.3)	10 (15.7)		
Smoking					
Yes	41	34 (82.9)	7 (17.1)	0.10	0.755
No	41	36 (87.8)	5 (12.2)		
Alcohol					
Yes	36	30 (83.3)	6 (16.7)	0.21	0.654
No	46	40 (87.0)	6 (13.0)		
Tumor location					
Upper	3	2 (66.7)	1 (33.3)	1.48	0.580
Middle	54	46 (85.2)	8 (14.8)		
Lower	25	22 (88.0)	3 (12.0)		
Gross appearance					
Polypoid	69	59 (85.5)	10 (14.5)	1.30	0.660
Ulcerative	8	6 (75.0)	2 (25.0)		
Infiltrating	5	5 (100.0)	0 (0.0)		
Lymph node metastasis					
Yes	23	21 (91.3)	2 (8.7)	0.36	0.547
No	59	49 (83.1)	10 (16.9)		
TNM stage					
I	29	27 (93.1)	2 (6.9)	4.40	0.121
II	37	28 (75.7)	9 (24.3)		
III	16	15 (93.8)	1 (6.2)		
IV	0	0 (0.0)	0 (0.0)

**Table 3:** The expression of AE1/AE3 protein in different components of PESC

	Number	Expression rate of AE1/AE3 protein (%)	Positive in epithelial component (%)	Positive in spindle component (%)	Positive in both of epithelial and spindle components (%)	*χ* ^*2*^	*P*
Age							
< 60 years	26	19 (73.1)	19 (73.1)	6 (23.1)	6 (23.1)	0.24	0.888
≥ 60 years	56	51 (91.1)	51 (91.1)	13 (23.2)	13 (23.2)		
Gender							
Male	57	48 (84.2)	48 (84.2)	13 (22.8)	13 (22.8)	0.00	1.000
Female	25	22 (88.0)	22 (88.0)	6 (24.0)	6 (24.0)		
Family history							
Positive	18	16 (88.9)	16 (88.9)	1 (5.6)	1 (5.6)	5.777	0.118
Negative	64	54 (84.3)	54 (84.3)	18 (28.1)	18 (28.1)		
Smoking							
Yes	41	34 (82.9)	34 (82.9)	10 (24.4)	10 (24.4)	0.197	0.978
No	41	36 (87.8)	36 (87.8)	9 (22.0)	9 (22.0)		
Alcohol							
Yes	36	30 (83.3)	30 (83.3)	10 (27.8)	10 (27.8)	1.154	0.764
No	46	40 (87.0)	40 (87.0)	9 (19.6)	9 (19.6)		
Tumor location							
Upper	3	2 (66.7)	2 (66.7)	1 (33.3)	1 (33.3)	6.42	0.126
Middle	54	46 (85.2)	46 (85.2)	16 (29.6)	16 (29.6)		
Lower	25	22 (88.0)	22 (88.0)	2 (8.0)	2 (8.0)		
Gross appearance							
Polypoid	69	59 (85.5)	59 (85.5)	15 (21.7)	15 (21.7)	3.22	0.794
Ulcerative	8	6 (75.0)	6 (75.0)	1 (12.5)	1 (12.5)		
Infiltrating	5	5 (100.0)	5 (100.0)	3 (60.0)	3 (60.0)		
Lymph node metastasis							
Yes	23	21 (91.3)	21 (91.3)	8 (34.8)	8 (34.8)	1.60	0.449
No	59	49 (83.1)	49 (83.1)	11 (18.6)	11 (18.6)		
TNM stage							
I	29	27 (93.1)	27 (93.1)	3 (10.3)	3 (10.3)	6.71	0.146
II	37	28 (75.7)	28 (75.7)	9 (24.3)	9 (24.3)		
III	16	15 (93.8)	15 (93.8)	7 (43.8)	7 (43.8)		
IV	0	0 (0.0)	0 (0.0)	0 (0.0)	0 (0.0)		

### CK5/6 expression and clinicopathological features

The positive CK5/6 staining was chiefly observed in cytoplasm of epithelial tumor cells, with a positive detection rate of 76.8% (63/82). CK5/6 protein was obviously expressed in age upper 60 years (*P* = 0.005). However, CK5/6 expression was not observed with any significant association with gender, family history, smoking, alcohol, tumor location, gross appearance, lymph node metastasis and TNM stage (*P* = 0.652, 0.671, 0.432, 0.382, 0.267, 0.350, 0.629, and 0.056, respectively, Table 4).

**Table 4:** The expression rate of CK5/6 protein in PESC

	Number	Expression rate of CK 5/6 protein (%)		*χ* ^*2*^	*P*
		Positive(*n* = 63)	Negative(*n* = 19)		
Age					
< 60 years	26	15 (57.7)	11 (42.3)	7.83	0.005
≥ 60 years	56	48 (85.7)	8 (14.3)		
Gender					
Male	57	43 (75.4)	14 (24.6)	0.20	0.652
Female	25	20 (80.0)	5 (20.0)		
Family history					
Positive	18	15 (83.3)	3 (16.7)	0.18	0.671
Negative	64	48 (75.0)	16 (25.0)		
Smoking					
Yes	41	30 (73.2)	11 (26.8)	0.62	0.432
No	41	33 (80.5)	8 (19.5)		
Alcohol					
Yes	36	26 (72.2)	10 (27.8)	0.77	0.382
No	46	37 (80.4)	9 (19.7)		
Tumor location					
Upper	3	2 (66.7)	1 (33.3)	2.87	0.267
Middle	54	39 (72.2)	15 (27.8)		
Lower	25	22 (88.0)	3 (12.0)		
Gross appearance					
Polypoid	69	53 (76.8)	16 (23.2)	2.08	0.350
Ulcerative	8	5 (62.5)	3 (37.5)		
Infiltrating	5	5 (100.0)	0 (0.0)		
Lymph node metastasis					
Yes	23	19 (82.6)	4 (17.4)	0.23	0.629
No	59	44 (74.6)	15 (25.4)		
TNM stage					
I	29	26 (89.7)	3 (10.3)	5.63	0.056
II	37	24 (64.9)	13 (35.1)		
III	16	13 (81.3)	3 (18.7)		
IV	0	0 (0.0)	0 (0.0)		

### Vimentin expression and clinicopathological features

**Fig. 3:**
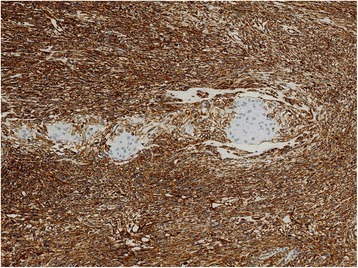
The protein expressions of vimentin in PESC tissues by immunohistochemistry (100×). The positive immunostaining of vimentin was localized in cell cytoplasm of spindle component

Vimentin positive expression was chiefly observed in the cytoplasm of spindle tumor cells. In this study, all the 82 patients (82/82, 100.0%) with vimentin were positive staining (Fig. 3). In contrast, only 7 PESC patients showed positive vimentin expression in epithelial tumor components. Moreover, the vimentin expression in different components of PESC did not show any correlation with age, gender, family history, smoking, alcohol, tumor location, gross appearance, lymph node metastasis, and TNM stage (*P* = 1.000, 1.000, 0.309, 1.000, 0.282, 0.832, 0.188, 1.000, and 0.067, respectively, Table 5).

**Table 5:** The expression of vimentin protein in different components of PESC

	Number	Expression rate of vimentin protein (%)	Positive in epithelial component (%)	Positive in spindle component (%)	Positive in both of epithelial and spindle components (%)	*χ* ^*2*^	*P*
Age							
< 60 years	26	26 (100.0)	2 (7.7)	26 (100.0)	2 (7.7)	0.15	1.000
≥ 60 years	56	56 (100.0)	5 (8.9)	56 (100.0)	5 (8.9)		
Gender							
Male	57	57 (100.0)	5 (8.8)	57 (100.0)	5 (8.8)	0.15	1.000
Female	25	25 (100.0)	2 (8.0)	25 (100.0)	2 (8.0)		
Family history							
Positive	18	18 (100.0)	3 (16.7)	18 (100.0)	3 (16.7)	3.347	0.309
Negative	64	64 (100.0)	4 (6.3)	64 (100.0)	4 (6.3)		
Smoking							
Yes	41	41 (100.0)	4 (9.8)	41 (100.0)	4 (9.8)	0.379	1.000
No	41	41 (100.0)	3 (7.3)	41 (100.0)	3 (7.3)		
Alcohol							
Yes	36	36 (100.0)	5 (13.9)	36 (100.0)	5 (13.9)	3.782	0.282
No	46	46 (100.0)	2 (4.3)	46 (100.0)	2 (4.3)		
Tumor location							
Upper	3	3 (100.0)	0 (0.0)	3 (100.0)	0 (0.0)	1.57	0.832
Middle	54	54 (100.0)	4 (7.4)	54 (100.0)	4 (7.4)		
Lower	25	25 (100.0)	3 (12.0)	25 (100.0)	3 (12.0)		
Gross appearance							
Polypoid	69	69 (100.0)	5 (7.2)	69 (100.0)	5 (7.2)	7.59	0.188
Ulcerative	8	8 (100.0)	0 (0.0)	8 (100.0)	0 (0.0)		
Infiltrating	5	5 (100.0)	2 (40.0)	5 (100.0)	2 (40.0)		
Lymph node metastasis							
Yes	23	23 (100.0)	2 (8.7)	23 (100.0)	2 (8.7)	0.21	1.000
No	59	59 (100.0)	5 (8.5)	59 (100.0)	5 (8.5)		
TNM stage							
I	29	29 (100.0)	0 (0.0)	29 (100.0)	0 (0.0)	7.50	0.067
II	37	37 (100.0)	5 (13.5)	37 (100.0)	5 (13.5)		
III	16	16 (100.0)	2 (12.5)	16 (100.0)	2 (12.5)		
IV	0	0 (0.0)	0 (0.0)	0 (0.0)	0 (0.0)		

### Predictive diagnostic model

**Fig. 4:**
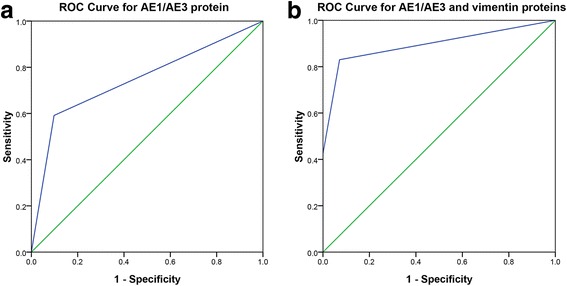
Predictive ability of diagnostic model. Predictive ability of diagnostic model compared with AE1/AE3 protein shown by receiver operating characteristic (ROC) curves (**a**) and area under the curve (AUC) in AE1/AE3 and vimentin proteins datasets (**b**)

To determine the diagnosis value with these two immunohistocheminal proteins in PESC, the predictive diagnostic model was calculated as Y = (β1) × (AE1/AE3) + (β2) × (vimentin), with Y equal to risk score and βn equal to each protein’s coefficient value from univariate cox proportional hazards regression analysis [8]. The corresponding coefficients were as follows: β1 = 0.337 and β2 = 0.519. Patients were ranked and divided into positive and negative groups using the 50th percentile (i.e., median) risk score as the cut-off value. The area under the receiver operating characteristic (ROC) curve for AE1/AE3 protein was 0.75 (95% CI = 0.68–0.82) (Fig. 4 A). In comparison with a single protein, the predictive power of the AE1/AE3 and vimentin proteins signature was increased apparently than with single signature [0.75 (95% CI = 0.68–0.82) with single protein in Fig. 3 A v.s. 0.89 (95% CI = 0.85–0.94) with AE1/AE3 and vimentin proteins in Fig. 4 B].

### CK protein expression and PESC survival

**Fig. 5:**
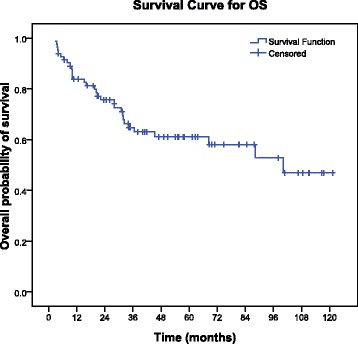
The curve for the whole cohort overall survival (OS)

**Fig. 6:**
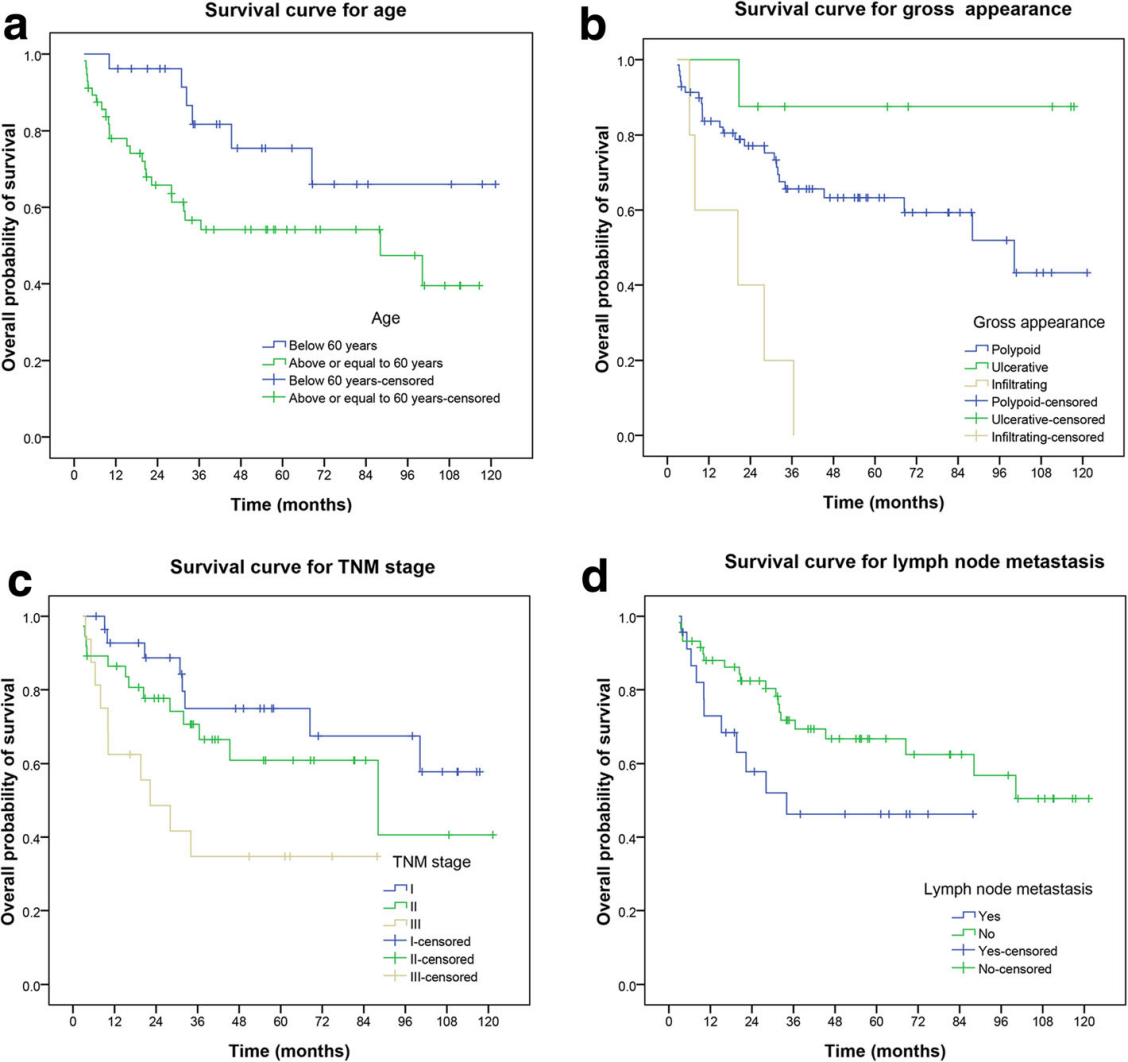
Kaplan-Meier analysis of overall survival for age (*P* = 0.036) (**a**), gross appearance (*P* = 0.001) (**b**), TNM stage (*P* = 0.015) (**c**) and lymph node metastasis (*P* = 0.072) (**d**) in the generation dataset of 82 cases

The median OS was 34.0 months (range, 2.9–121.3 months) (Fig. 5). The 1-, 3-, 5- and 7-year survival rates for PESC patients in this study were 79.3%, 46.3%, 28.0% and 15.9%, respectively. Kaplan-Meier analysis showed that age, gross appearance and TNM stage were the important factors to affect survival. The PESC patients with age under 60 years had a better survival than age upper 60 years (*P* = 0.036, Fig. 6 A). The survival in the infiltrating gross appearance was of the worst prognosis than other gross appearances (*P* = 0.001, Fig. 6 B). The TNM stage used for esophageal squamous cell carcinoma could make a clear different survival curve for stage I, II and III (*P* = 0.015, Fig. 6 C). Furthermore, multivariate analysis demonstrated that age and TNM stage were independent prognostic factors for overall survival (P = 0.036 and 0.003, respectively, Table 6). Surprisingly, the univariate analyses showed no difference for survival in the patients with and without lymph node metastasis (0.51 (95% CI = 0.23–1.08), *P* = 0.072, Fig. 6 D). And there was no significant difference among lymph node metastasis and 1-,3-, 5-, 7- and over 7-year survival (*P* = 0.129, Table 7). Similar results were observed in tumor location (*P* = 0.109), gender (*P* = 0.537), smoking (*P* = 0.348), alcohol (*P* = 0.850), and family history (*P* = 0.115).

**Table 6:** Univariate and multivariate analysis of survival on clinicopathological factors

	Number	Univariate analysis			Multivariate analysis		
		HR	95% CI	*P*	HR	95% CI	*P*
Age							
< 60 years	26	2.52	(1.03–6.16)	0.036	2.66	(1.06–6.63)	0.036
≥ 60 years	56						
Gender							
Male	57	0.77	(0.34–1.75)	0.537			
Female	25						
Family history							
Positive	18	0.40	(0.12–1.31)	0.115			
Negative	64						
Smoking							
Yes	41	0.71	(0.35–1.46)	0.348			
No	41						
Alcohol							
Yes	36	1.07	(0.53–2.18)	0.850			
No	46						
Tumor location							
Upper	3	1.96	(0.96–4.01)	0.109			
Middle	54						
Lower	25						
Gross appearance							
Polypoid	69	1.58	(0.90–2.77)	0.001	1.38	(0.79–2.39)	0.256
Ulcerative	8						
Infiltrating	5						
Lymph node metastasis							
Yes	23	0.51	(0.24–1.08)	0.072			
No	59						
TNM stage							
I	29	1.95	(1.18–3.21)	0.015	2.11	(1.28–3.50)	0.003
II	37						
III	16						
IV	0						
AE1/AE3 protein							
Positive	70	1.10	(0.83–1.45)	0.527			
Negative	12						
CK5/6 protein							
Positive	63	1.82	(0.70–4.73)	0.222			
Negative	19						

*Abbreviations*: *CI* confidence interval, *HR* hazard ratio

**Table 7:** Lymph node metastasis and survival

Survival (year)	Lymph node metastasis		*χ* ^*2*^	P
	Yes (*n* = 23)	No (*n* = 59)		
1	16 (69.5)	49 (83.1)	6.697	0.129
3	6 (26.1)	30 (50.8)		
5	2 (8.7)	17 (28.8)		
7	0 (0.0)	12 (20.3)		
Over 7	0 (0.0)	10 (16.9)		

The univariate and multivariate analyses showed that AE1/AE3 and CK5/6 proteins expression were not related with PESC survival (Table 6).

### Comparison on PESC diagnosis by biopsy with surgical resection specimen

In the present study, all the 82 patients were confirmed as PESC by surgical pathology. However, only 14 patients had a PESC diagnosis by biopsy pathology before surgery (14/82, 17.1%). Of the misdiagnosed patients with biopsy pathology, 72.4% had been diagnosed as squamous cell carcinoma followed by adenocarcinoma and others.

### Clinicopathological features with different stage

In this study, most of PESC symptoms at presentation were dysphagia, (93.1% in stage I, 86.5% in stage II, and 93.8% in stage III), followed by odynophagia (6.9% in stage I, 5.4% in stage II and 6.2% in stage III), and no difference was observed for the early (stage I) and advanced (stage II and III) in symptom distribution (*P* = 0.594). Similarly, it did not show any correlation for the staging with age (*P* = 0.494), gender (*P* = 0.911), family history (*P* = 0.885), smoking (*P* = 0.768), alcohol (*P* = 0.242), tumor location (*P* = 0.960) and gross appearance (*P* = 0.221, Table 8).

**Table 8:** The clinicopathological features with different stage in PESC

	Number	Stage (%)			*χ* ^*2*^	*P*
		I(*n* = 29)	II(*n* = 37)	III(*n* = 16)		
Age						
< 60 years	26	7 (26.9)	14 (53.8)	5 (19.3)	1.41	0.494
≥ 60 years	56	22 (39.3)	23 (41.1)	11 (19.6)		
Gender						
Male	57	21 (36.8)	25 (43.9)	11 (19.3)	0.19	0.911
Female	25	8 (32.0)	12 (48.0)	5 (20.0)		
Family history						
Positive	18	6 (33.3)	9 (50.0)	3 (16.7)	0.25	0.885
Negative	64	23 (35.9)	28 (43.8)	13 (20.3)		
Smoking						
Yes	41	15 (36.6)	17 (41.5)	9 (21.9)	0.53	0.768
No	41	14 (34.1)	20 (48.8)	7 (17.1)		
Alcohol						
Yes	36	11 (30.6)	15 (41.7)	10 (27.7)	2.84	0.242
No	46	18 (39.1)	22 (47.8)	6 (13.1)		
Tumor location						
Upper	3	1 (33.3)	2 (66.7)	0 (0.0)	1.17	0.960
Middle	54	19 (35.2)	23 (42.6)	12 (22.2)		
Lower	25	9 (36.0)	12 (48.0)	4 (16.0)		
Gross appearance						
Polypoid	69	25 (36.2)	30 (43.5)	14 (20.3)	5.21	0.221
Ulcerative	8	4 (50.0)	4 (50.0)	0 (0.0)		
Infiltrating	5	0 (0.0)	3 (60.0)	2 (40.0)		
Lymph node metastasis						
Yes	23	1 (4.3)	7 (30.4)	15 (65.3)	42.27	0.000
No	59	28 (47.5)	30 (50.8)	1 (1.7)		

## Discussion

As we knew, this is the largest sample size report on cytokeratin and vimentin protein expression and PESC diagnosis and prognosis. The present results demonstrate that cytokertain, expressed chiefly in epithelial tumor cells, and vimentin, expressed always in spindle tumor cells, are useful biomarker in PESC diagnosis, especially, the predictive power of the AE1/AE3 and vimment proteins signature together was increased apparently than with single signature. However, the AE1/AE3, CK5/6 and vimment proteins expressions did not show any significant effects on PESC survival. Furthermore, no relationship was observed for the AE1/AE3 and vimment proteins expression and age, gender, tumor location, gross appearance, lymph node metastasis, and TNM stage. Similarly, the expression CK5/6 did not show relationship with gender, tumor location, gross appearance, lymph node metastasis, and TNM stage. The major genetic abnormalities for PESC remain largely unknown. Only few series genetic studies have been published with conflicting results regarding the type of alteration present in the two tumor components. The p53 protein over-expression and CD-1 gene amplification seem to occur frequently in both tumor components, in contrast, E-cadherin protein expression is observed chiefly in epithelial component [9, 10]. However, because of that most genetic studies involve single or small number of cases, the value of the observed genetic changes in PESC prognosis is not clear.

Interestingly, the present study demonstrates that 23.2% of PESC has a positive AE1/AE3 immunoreactivity in both epithelial and spindle tumor components. Similar results are observed in 8.5% of PESC for vimentin. These findings suggest that PESC may originate from same clone. The histogenesis for PSEC remains inconclusive. Further whole genomic sequencing pattern may shed light on the molecular clue for PESC histogenesis.

The present study demonstrates a slight better 5-year survival for PSEC than esophageal squamous cell carcinoma [11]. The gross appearance and TNM stage are independent prognostic factors for overall survival, and, in this study, the PESC patients with lymph node metastasis did not show worsen prognosis than those without lymph node metastasis. The reason is not clear. Lymph node metastasis has been well documented as risk factor for esophageal squamous cell carcinoma [12–17]. It is noteworthy that the prevalence of lymph node metastasis in PESC is apparently lower than in esophageal squamous cell carcinoma, which may contribute to the better survival for PESC than the esophageal squamous cell carcinoma. Histopathologically, PESC is composed by epithelial and spindle components. In the present study, only 23 PSEC patients occurring lymph node metastasis, almost all the lymph node metastatic components are epithelial. Therefore, it is desirable to characterize the PESC lymph node metastasis by different components to correlate with survival in large cohort study.

It is noteworthy that the biopsy accurate diagnosis for PESC before radical esophagectomy is much lower than the surgical diagnosis. 72% of PESC had been diagnosed as squamous cell carcinoma. Obviously, the too small size is the major reason for this poor biopsy accurate diagnosis. Another reason is because of the predominant epithelial component in these PESC patients. Fortunately, this partial diagnosis has no impact on therapy for the moment [9].

## Conclusions

In summary, the present study demonstrates that cytokeratin and vimentin protein immunoassay is a useful biomarker for PESC accurate diagnosis, but not prognosis. The co-expression of cytokeratin and vimentin in both epithelial and spindle components suggest the possibility of single clone origination for PESC. PESC occurs predominantly in male patient (male:female, 2.3:1) with a peak age of 60 years old. PESC are located more frequently in the middle segment. The age and TNM stage of PESC are independent prognostic factors.

## Abbreviations

AUC: Area under the curve
CI: Confidence interval
CK: Cytokeratin
DAB: Diaminobenzidine
HR: Hazard ratio
PESC: Primary esophageal spindle cell carcinoma
ROC: Receiver operating characteristic
